# Fear of Recurrence and Progression in People with Heart Disease: Risk Factors and Implications for Emotional Support

**DOI:** 10.3390/bs15040479

**Published:** 2025-04-06

**Authors:** Sarah T. Clarke, Barbara M. Murphy, Robert Hester, Alun C. Jackson

**Affiliations:** 1 Australian Centre for Heart Health, Melbourne, VIC 3051, Australia; 2 Melbourne School of Psychological Sciences, University of Melbourne, Parkville, VIC 3052, Australia; 3 Centre on Behavioral Health, University of Hong Kong, Hong Kong SAR, China

**Keywords:** fear of progression, fear of recurrence, fear of recurrence and progression, cardiac distress, illness uncertainty

## Abstract

Support to manage fear of recurrence and progression (FoRP) is a major concern and a commonly unmet need for people with chronic illness. The current study identified profiles of and risk factors for FoRP in people with heart disease. A sample of 241 participants completed 44 cardiac-specific FoRP items and provided demographic, clinical, and psychosocial information. Cluster analysis identified three profiles: a high-, moderate-, and low-FoRP group. Patients who were younger, had a comorbid health condition(s), and higher levels of uncertainty and cardiac-related distress were at the most risk of higher FoRP. By characterizing the nature and correlates of cardiac-FoRP, this study enables health professionals to understand the specific concerns of their patients and assists in identifying those at greatest risk. The findings extend the emerging field of cardiac-FoRP research and will assist in the development of a cardiac-specific screening measure and of tailored and targeted interventions to support cardiac patients in their emotional recovery.

## 1. Introduction

Living with heart disease is inherently distressing due to a combination of debilitating physical symptoms, restrictions in daily social and role functioning, and uncertainty about the future (Jackson et al., 2022b). Regarding future concerns specifically, fear of disease recurrence and progression have been shown to be both common and distressing for people with heart disease (Clarke et al., 2024b; Herwig et al., 2019; Iles-Smith et al., 2017; Withers et al., 2015). A recent study demonstrated that over 40% of cardiac patients experience fear of disease recurrence or progression, and one in two experience moderate to severe distress due to such fears (Clarke et al., 2024b). Living with fear can impact clinical outcomes for cardiac patients, with studies demonstrating that greater fear of dying is associated with increased cardiac risk (von Känel et al., 2011) and can trigger depression and anxiety (Whitehead et al., 2005).

There is a well-established body of research investigating fear of disease recurrence and progression (FoRP) in chronic illnesses more broadly. FoRP is a transdiagnostic construct used to describe the fear that a disease will recur or progress, with all related consequences (Herschbach et al., 2005; Herschbach & Dinkel, 2014; Sharpe et al., 2022). The construct was introduced as a framework to describe disease-specific distress in chronic illness patients that could not be appropriately described as anxiety in that the fears represent an appropriate response to the real threats of one’s disease experience rather than being excessive or out of proportion (Herschbach et al., 2005). Most research has been in the field of oncology, where over a decade ago, FoRP was described as one of patients’ top concerns and the most unmet support need (Simard et al., 2013). Since that time, numerous studies have investigated the prevalence, predictors, and impacts of FoRP in cancer and other patient groups (Calderon et al., 2024; Folkerts et al., 2022; Lebel et al., 2020; Luigjes-Huizer et al., 2022; Pang & Humphris, 2021; Smith et al., 2020). More recently, numerous psychosocial interventions have been designed to assist in the management of FoRP for cancer patients (Kang & Yu, 2023; Liu et al., 2019; Tauber et al., 2019).

In contrast, research into cardiac-FoRP is in its infancy. Only a handful of studies have attempted to characterize cardiac-FoRP, with findings suggesting that cardiac-FoRP may be associated with cardiac event-induced post-traumatic stress disorder (PTSD) (Fait et al., 2018), social support, disease course, and recurrences in coronary heart disease patients (Zhen et al., 2022) and symptom perception, self-care confidence, resilience, and sleep quality in chronic heart failure (Xiong et al., 2023a, 2023b). While these studies provide insight into the impacts of cardiac-FoRP, they do not identify cardiac patients’ specific concerns regarding FoRP, nor do they identify the profiles of patients most at risk. Further research is required to understand the specific FoRP-related issues experienced by people living with a heart condition and to identify the risk factors for cardiac-FoRP.

An additional limitation of cardiac-FoRP research has been the use of FoRP outcome measures that have not been validated in cardiac populations. Having been designed for use in other chronic illness groups, these measures include items that are less relevant to cardiac-FoRP and omit others that are central (Clarke et al., 2024b). FoRP is multidimensional, involving a range of different fears that vary between individuals with different disease experiences and can evoke both adaptive and maladaptive behavioral responses (Almeida et al., 2019; Clarke et al., 2024b; Lebel et al., 2016). To measure and address FoRP in cardiac patients, it is critical to comprehensively characterize specific cardiac-FoRP-related issues and evolve beyond utilizing frameworks and measures developed from different illness experiences.

We developed a preliminary conceptual framework for cardiac-FoRP through a scoping review of qualitative studies investigating psychosocial concerns in people living with heart disease (Clarke et al., 2024b). Several broad domains of cardiac-FoRP were identified, including concerns around one’s health, dying, relationships, treatment, accessing help, roles and responsibilities, and physical activity. Common behavioral responses when patients were confronted with cardiac-FoRP included avoidance, hyperawareness, symptom misattribution, seeking help, and making lifestyle changes (Clarke et al., 2024b). Further research is required to refine this framework and understand how cardiac-FoRP differs between individuals.

The current study aimed to further characterize the nature and correlates of cardiac-FoRP by (a) investigating the endorsement of a range of cardiac-FoRP issues and domains, (b) identifying sub-groups of cardiac-FoRP, and (c) identifying the demographic, psychosocial, and clinical correlates of each sub-group of cardiac-FoRP. By developing a deeper understanding of the specific nature and correlates of FoRP in people living with heart disease, the objective of the present study was to assist health professionals in the identification and management of cardiac-FoRP.

## 2. Materials and Methods

### 2.1. Participants

Eligible participants were adults who have had an acute coronary event, undergone cardiac surgery, or had a chronic cardiac condition. Participants were excluded if they did not have adequate English language proficiency to read and understand the Participant Information and Consent Form (PICF) and questionnaire. Participants self-selected to participate in the study. The study was promoted through hard-copy flyers in healthcare settings, social media posts, and other online communications.

### 2.2. Materials

#### 2.2.1. Cardiac-FoRP Items

The items used to investigate cardiac-FoRP were developed through (i) reviewing both the literature and existing measures and (ii) consultation with and feedback from key informants. This process has been outlined in detail elsewhere (Clarke et al., 2024a). Briefly, cardiac-FoRP was initially conceptualized through reviewing the existing literature in both cardiology and other areas of chronic illness research. In particular, a scoping review of qualitative studies identified key concerns relating to fear of recurrence and progression in cardiac patients (Clarke et al., 2024b). The initial item pool was developed through both adapting relevant items from existing FoRP scales and existing measures of anxiety, fear, and distress in cardiac patients, and by creating new items regarding concerns that were identified within the scoping review. Generated items were reworded where appropriate to ensure relevance to the measurement of cardiac-FoRP and appropriateness of fit with the instruction and response set that followed. The generated items underwent a collaborative, iterative validation process through which three stages of key informants (stage 1: health professionals, stage 2: academic experts, stage 3: cardiac patients) provided feedback and suggestions on the item pool.

The wording of the stem, items, and responses was developed from a combination of reviewing existing measures, key informants, and multidisciplinary group decisions. The general instructions read: “*Living with a heart condition can sometimes be difficult. People often worry about their life and the future. Below is a list of concerns you may have about your condition becoming worse or having another heart event. Please indicate how concerned you are about each of the issues listed below on a scale of 0 to 3. If the statement does not relate to you, please choose “not at all”*”. Items were separated into two groups, which required different responses/stems, depending on whether they were addressing a fear or a response to fear. For fear items, the stem was “*in regard to your heart condition, how concerned are you about…”.* Responses were on a four-point Likert scale, including zero = not at all, one = slightly, two = moderately, and three = extremely. For behavioral items, the stem was *“Because of your concerns about having another heart event or your condition getting worse, how often do you*…”. Responses were on a four-point Likert scale, including zero = not at all, one = a little of the time, two = some of the time, and three = a lot of the time.

#### 2.2.2. Additional Measures

All information was self-reported. *Demographic information* collected included age, sex, country of residence and birth, marital/partner status, employment status, and private health insurance status. In Australia, having private health cover is associated with outright home ownership, luxury vehicle ownership, and a six-figure income (Muggli et al., 2006), hence its relevance as an indicator of financial security. *Psychosocial information* collected included whether participants lived alone, had a close confidante (someone they could talk to about how they are feeling), had a recent bereavement (lost an important relative or friend in the past 12 months), were experiencing financial strain (dichotomized as either none/slight or moderate/considerable/extreme), had work impacted by their cardiac condition (stopped working, reduced hours at work, or changed jobs), and had a history of mental ill-health (diagnosis of anxiety, depression, PTSD, or other mental health conditions). *Clinical information* collected included types of cardiac conditions, events, and procedures from a list of options including heart attack/acute coronary syndrome (ACS), coronary artery bypass graft surgery (CABGS), percutaneous coronary intervention (PCI), spontaneous coronary artery dissection (SCAD), cardiac arrest, valve surgery, heart rhythm disturbance, heart failure, congenital heart disease, takotsubo cardiomyopathy, or the option to specify other. Clinical information also included the number of cardiac events and/or diagnoses and/or procedures, presence of cardiac risk factors (high blood pressure, high cholesterol, obesity, cigarette smoking, diabetes), presence of comorbid health condition(s), cardiac rehabilitation attendance, and whether they had received mental health support following their cardiac event/diagnosis/procedure (including face-to-face, online, individual, and group supports). The 7-item *Generalised Anxiety Disorder Instrument (GAD-7)* (Spitzer et al., 2006), which has been validated for use in cardiac populations (Conway et al., 2016), was used to measure anxiety symptoms. The 9-item *Patient Health Questionnaire-9 (PHQ-9)* (Kroenke et al., 2001), which has been validated with cardiac patients (Stafford et al., 2007; Thombs et al., 2008), was used to measure depression symptoms. Cardiac distress was measured using the 12-item *Cardiac Distress Inventory Short Form* (Le Grande et al., 2023) *(CDI-SF)*, which has excellent internal consistency and good convergent and discriminant validity (Le Grande et al., 2023). The 20-item *PTSD Checklist for DSM-5 (PCL-5)* (Weathers et al., 2013) assessed symptoms of PTSD. Participants were asked to answer questions specifically in relation to their cardiac event, diagnosis, or procedure. Illness uncertainty was measured using the 23-item *Mishel Uncertainty in Illness Scale–Community Form (MUIS-C)* (Mishel, 1981), designed to assess uncertainty in illness in non-hospitalized chronically ill people. An adapted 18-item form of the *Metacognitions Questionnaire-30 (MCQ-30)* (Wells & Cartwright-Hatton, 2004) was used to assess metacognitive beliefs. The adapted version, which has been used previously in a study of FoRP in cancer (Coutts-Bain et al., 2022), comprises three of the original five subscales that are more highly associated with FoRP (Butow et al., 2015; Thewes et al., 2013). These subscales are positive beliefs about worry (e.g., *worrying helps me to avoid problems in the future*), negative beliefs about worry (e.g., *my worrying is dangerous for me*), and need for control over thoughts (e.g., *if I did not control a worrying thought, and then it happened, it would be my fault*).

### 2.3. Procedure

Participants completed the PICF and questionnaire pack online. Access to the online questionnaire was obtained by scanning a QR code on physical flyers or by following the link from online posts. In instances where participants were unable to access a device or the internet to complete the measures, they were provided a hard-copy questionnaire pack (*n* = 5). Access to the hard-copy questionnaire was obtained by post. No identifying information (name, address, date of birth) was collected.

### 2.4. Statistical Analysis

All statistical analyses were completed on IBM SPSS (v. 29) (IBM Corp., 2023) and Jamovi (v. 2.6.2) (The Jamovi Project, 2024). Data were cleaned to remove duplicates identified based on identical demographic information. In the case of duplicates, the first entry was retained unless the participant reported further cardiac events or additional diagnoses from the original entry, in which case, the most recent entry was retained, resulting in 29 cases removed. Cases with missing data on the 44 cardiac-FoRP items were removed from analysis (*n* = 4).

Frequencies were calculated for the endorsement rate of each of the 44 cardiac-FoRP items. The items were grouped into seven pre-determined domains (five fear domains and two behavioral domains) based on the conceptual framework (Clarke et al., 2024b) and the item generation process described above. The fear domains included the following: health (six items), interpersonal (seven items), self (seven items), treatment (seven items), and roles (four items). The behavioral domains included the following: avoidance (eight items) and hyperawareness (five items).

Cluster analysis identified participants who experienced similar patterns of FoRP across the seven domains. The ideal number of clusters was investigated using hierarchical cluster analysis, consulting the scree plot of distances from the agglomeration schedule and dendrogram using Ward’s method and squared Euclidean distance. The ideal number of clusters was then validated using the variance ratio criterion to calculate the *ω_k_* for three- to five-factor solutions, where the lowest *ω_k_* indicates the optimal solution (Sarstedt & Mooi, 2019). K-means cluster analysis was subsequently used to establish membership of the number of clusters established from the hierarchical analysis (Sarstedt & Mooi, 2019).

Chi-square analyses and analyses of variance (ANOVA) were used to examine differences in demographic and psychosocial outcomes between the FoRP clusters. In the chi-square analyses between different cardiac conditions, patients with SCAD (*n* = 8), Takostubo cardiomyopathy (*n* = 4), or who specified another condition (see Table S1; *n* = 24) were grouped together to form an ‘other’ group, as the rates for each condition were too low to conduct between-group investigation. Since normality was violated for all continuous variables investigated, the Kruskal–Wallis analysis test was used for ANOVAs, reporting *ε*^2^ for effect size, using the Dwass–Steel–Critchlow–Fligner (DSCF) test as a non-parametric pairwise comparison.

Ordinal logistical regression was used to identify the associates of membership in different FoRP groups. Selection of items to be entered into the logistic regression analysis was based on statistical significance at the level of *p* ≤ 0.2 in the bivariate analyses to minimize the possibility of Type 2 error. Due to the large number of cardiac conditions, these variables were not considered for inclusion in the logistic regression. Variables were assessed for cases of significant collinearity with another variable, as determined through a variance inflation factor (VIF) greater than five.

## 3. Results

### 3.1. Sociodemographic and Medical Characteristics

Participants (121M, 120F) were aged between 26 and 89 (*M* = 64.52, *SD* = 11.23). Most were born in Australia (*n* = 174, 72%), the United Kingdom (UK, *n* = 26, 11%), or New Zealand (NZ, *n* = 12, 5%). The remaining 29 participants (12%) were born in a wide range of countries spanning across Europe (*n* = 10), Asia (*n* = 7), North America (*n* = 7), Africa (*n* = 4), and Oceania (*n* = 1). Most participants resided in Australia (*n* = 234, 97%), with the remaining living in the UK (*n* = 2), the United States of America (*n* = 2), NZ (*n* = 2), and Singapore (*n* = 1). Participants were recruited through their health service (*n* = 142, 59%), from social media or online (*n* = 64, 27%), and other avenues (*n* = 35, 14%), including previous research participants and word of mouth referrals.

The most common cardiac conditions, events, and/or procedures were ACS/acute myocardial infarction (AMI) (39%), heart rhythm disturbance (37%), PCI (33%), CAGBS (26%), valve surgery (19%), and heart failure (16%). Of those who provided data (*n* = 229), time since first event or diagnosis ranged from 1 week to 47 years prior to study completion, with a mean time of 5.8 years (*SD* = 9.0 years). Of those who provided data (*n* = 225), time since the most recent event or diagnosis ranged from 1 week to 25 years prior to study completion, with a mean time of 1.8 years (*SD* = 3.1 years). Other clinical and demographic information is presented in Table 1.

### 3.2. Fears Relating to Cardiac Disease Progression and Recurrence

**Figure 1:**
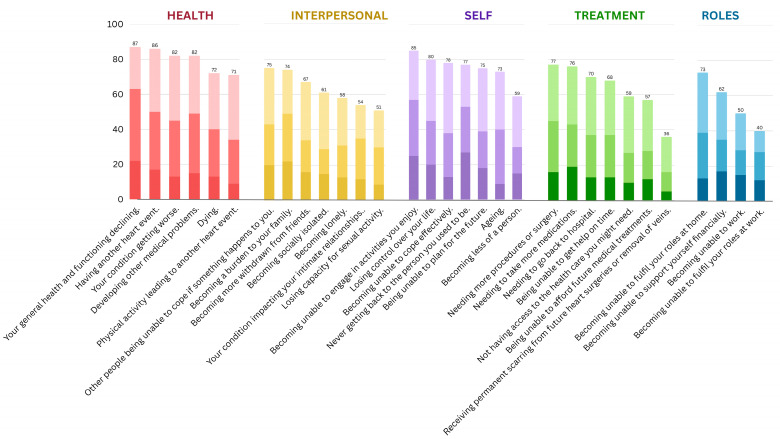
Endorsement rates of FoRP concerns by domains. Darkest to lightest shades represent ‘extreme’, ‘moderate’, and ‘slight’ concern, respectively, about each item.

**Figure 2:**
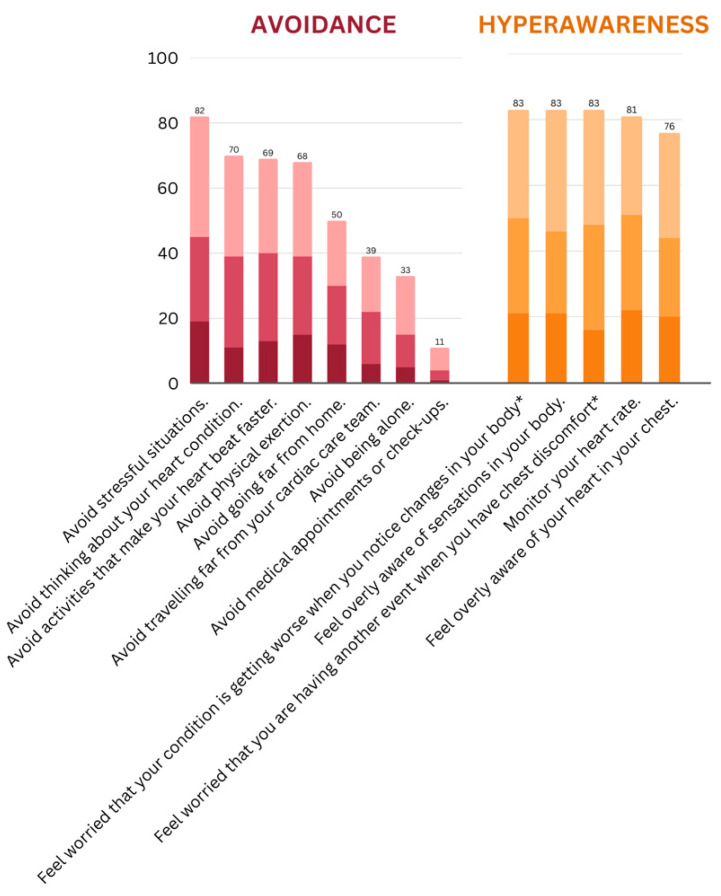
Endorsement rates of FoRP behaviors by domains. Darkest to lightest shades represent ‘a little of the time, ‘some of the time’, and ‘a lot of the time’, respectively, about each behavior. * Shortened version of items presented in the graph.

Ratings for each item across the five fear domains are depicted in Figure 1. Ratings across the two behavioral domains are depicted in Figure 2. The endorsement rates for each item are also provided in Table S2. Overall endorsement rates of fears were high across the sample, with an average of 80% endorsement across the health domain, 75% across the self domain, 63% across the interpersonal and treatment domains, and 57% across the role domain. The most highly endorsed fears included ‘*your general health and functioning declining*’, ‘*having another heart event*’, and ‘*becoming unable to engage in activities you enjoy*’. Behaviors relating to FoRP were also highly endorsed, with hyperawareness endorsed by 81% of the sample and avoidance in 53% of the sample. The most highly endorsed behaviors included ‘*feel overly aware of sensations in your body*’, ‘*feel worried that you are having another event when you have chest discomfort, or when your heartbeat is fast or irregular*’, and ‘*feel worried that your condition is getting worse when you notice changes in your body, such as feeling more fatigued, short of breath, or retaining more fluid*’.

In terms of severity, the percentage of participants rating being ‘extremely’ concerned about items was, on average, 18% in the self domain, 17% in the interpersonal domain, 15% in the health domain, 14% in the role domain, and 13% in the treatment domain. The fears most highly endorsed as ‘*extremely*’ concerning included ‘*never getting back to the person you used to be*’ (27% extremely concerned), ‘*becoming unable to engage in activities you enjoy*’ (25% extremely concerned), and ‘*your general health and functioning declining*’ (22% extremely concerned). The percentage of participants who rated engaging in behaviors ‘*a lot of the time*’ was, on average, 20% for hyperawareness and 10% for avoidance behaviors. The behaviors most highly endorsed as ‘*a lot of the time*’ included ‘*monitor your heart rate*’ (22% a lot of the time), ‘*feel overly aware of sensations in your body*’ (21% a lot of the time), and ‘*feel worried that your condition is getting worse when you notice changes in your body, such as feeling more fatigued, short of breath, or retaining more fluid*’ (21% a lot of the time).

### 3.3. Fear of Progression and Recurrence Profiles and Characteristics of Each Profile

**Figure 3:**
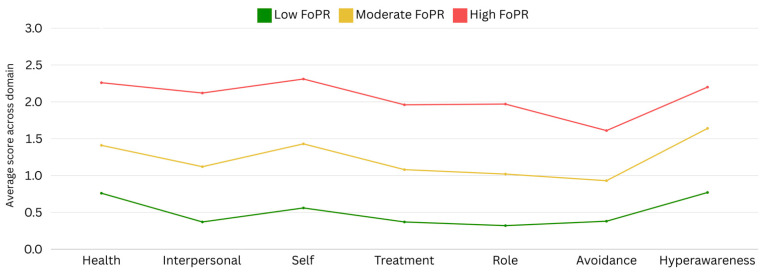
Results of cluster analysis across FoRP domains.

Cluster analysis identified three profiles: a high-fear (*n* = 62, 26%), moderate-fear (*n* = 100, 41%), and low-fear (*n* = 79, 33%) group (Figure 3).

In chi-square analyses (Table 1), the high-FoRP group was associated with being female, not having health insurance, having no close confidante, experiencing moderate-to-severe financial strain, having work impacted by ones cardiac condition, having a comorbid health condition(s), and having a history of a mental health condition, whereas the opposite of these was associated with being in the low-FoRP group. Those in the low-FoRP group were also more likely to have only a single cardiac event or diagnosis. Those in the high-FoRP group were more likely to have a mid-level (trade) education and less likely to have a university education, while the opposite was true for those in the moderate-FoRP group. Those with heart rhythm disturbances were more likely to be in the moderate-FoRP group and less likely to be in the low-FoRP group, whereas those with heart failure were more likely to be in the high-FoRP group and less likely to be in the moderate-FoRP group. Those with ‘other’ cardiac disorders were more likely to be in the high-FoRP group and less likely to be in the low-FoRP group.

No relationship was seen between FoRP profile and partnership status, employment status, living alone, having recent bereavement, having cardiac risk factors, cardiac rehabilitation attendance, and receiving mental health support following cardiac event/diagnosis. No differences in FoRP profiles were seen between those with ACS, CAGBS, valve surgery, PCI, cardiac arrest, and congenital heart disease.

**Table 1:** Differences in participant characteristics across FoRP profiles (chi-square analyses).

	*N*	Low FoRP *n* (%)	Moderate FoRP *n* (%)	High FoRP *n* (%)	*X* ^2^	*df*	*p*
Overall	241	79 (33)	100 (41)	62 (26)			
**Demographic**							
Gender					7.78	2	0.02
Female	120	31 (**26**)	50 (42)	39 (**33**)			
Male	121	48 (**40**)	50 (41)	23 (**19**)			
Partnered					0.61	2	0.74
Yes	172	59 (34)	71 (41)	42 (24)			
No	68	20 (29)	29 (43)	19 (28)			
Employment status					0.05	2	0.98
In paid employment	96	32 (33)	40 (42)	24 (25)			
Not in paid employment	145	47 (32)	60 (41)	38 (26)			
Education					15.78	4	0.003
≤Secondary	44	17 (39)	14 (32)	13 (30)			
Mid-level (trade)	59	21 (36)	15 (**25**)	23 (**39**)			
University	138	41 (30)	71 (**51**)	26 (**19**)			
Health insurance					18.88	2	<0.001
Yes	178	66 (**37**)	79 (44)	33 (**19**)			
No	63	13 (**21**)	21 (33)	29 (**46**)			
**Psychosocial**							
Live alone					1.98	2	0.37
Yes	55	17 (31)	27 (49)	11 (20)			
No	186	62 (33)	73 (39)	51 (27)			
Close confidante					10.75	2	0.005
Yes	204	73 (**36**)	86 (42)	45 (**22**)			
No	37	6 (**16**)	14 (38)	17 (**46**)			
Recent bereavement							
Yes	86	25 (29)	36 (42)	25 (29)	1.15	2	0.55
No	155	54 (35)	64 (41)	37 (24)			
Moderate to extreme financial strain							
Yes	89	18 (**20**)	33 (37)	38 (**43**)	23.25	2	<0.001
No	152	61 (**40**)	67 (44)	24 (**16**)			
Worked impacted by cardiac health							
Yes	101	19 (**19**)	47 (47)	35 (**35**)	16.97	2	<0.001
No	139	60 (**43**)	52 (37)	27 (**19**)			
Mental health history							
Yes	94	23 (**24**)	40 (43)	31 (**33**)	6.22	2	0.04
No	147	56 (**38**)	60 (41)	31 (**21**)			
**Clinical**							
Cardiac condition/diagnosis							
ACS/Heart attack	94	31 (33)	38 (40)	25 (27)	0.09	2	0.96
Bypass surgery	62	23 (37)	23 (37)	16 (26)	0.86	2	0.65
Valve surgery	46	17 (37)	22 (48)	7 (15)	3.29	2	0.19
Stent/PCI	79	33 (42)	27 (34)	19 (24)	4.45	2	0.10
Cardiac arrest	18	5 (28)	7 (39)	6 (33)	0.62	2	0.73
Heart rhythm disturbance	88	20 (**23**)	44 (**50**)	24 (27)	6.82	2	0.03
Heart failure	39	9 (23)	10 (**26**)	20 (**51**)	15.97	2	<0.001
Congenital heart disease	21	4 (19)	10 (48)	7 (33)	2.05	2	0.36
Other	36	3 (**8**)	17 (48)	16 (**44**)	13.81	2	0.001
More than 1 event/diagnosis							
Yes	139	36 (**26**)	63 (45)	40 (29)	6.96	2	0.03
No	100	42 (**42**)	37 (37)	21 (21)			
Cardiac risk factors ^a^							
Any	158	50 (32)	64 (41)	44 (28)	1.09	2	0.58
None	83	29 (35)	36 (43)	18 (22)			
Comorbidity ^b^					8.86	2	0.01
Yes	152	42 (**28**)	62 (41)	48 (**32**)			
No	89	37 (**42**)	38 (43)	14 (**16**)			
Attend cardiac rehab					3.18	2	0.20
Yes	190	61 (32)	84 (44)	45 (24)			
No	51	18 (35)	16 (31)	17 (33)			
Mental health support following cardiac event/condition							
Yes	110	31 (28)	50 (45)	29 (26)	2.10	2	0.35
No	131	48 (37)	50 (38)	33 (25)			

^a^ Cardiac risk factors included high blood pressure (*n* = 112, 46%), high cholesterol (*n* = 84, 35%), obesity (*n* = 45, 19%), cigarette smoking (*n* = 7, 3%), and diabetes (*n* = 38, 16%). ^b^ The top 5 comorbidities included musculoskeletal disease (26% of sample), obesity (19%), sleep apnea (18%), diabetes (16%), and cancer (8%). FoRP = fear of recurrence or progression; ACS = acute coronary syndrome; PCI = percutaneous coronary intervention. Bolded numbers reflect where the adjusted standardized residuals were greater than +/− 1.96.

In ANOVA (Table 2), higher FoRP was significantly associated with younger age, and higher scores for anxiety, depression, PTSD, cardiac distress, illness uncertainty, positive beliefs, negative beliefs, and need for control. Post hoc pairwise comparisons showed that each of the three FoRP groups differed significantly for almost all comparisons, with only a few exceptions, as shown in Table 3. Specifically, the low and moderate FoRP groups did not differ significantly in terms of age, while the moderate and high FoRP groups did not differ significantly in terms of either positive beliefs or need for control. There were no differences in terms of time since the most recent or first cardiac event/diagnosis/procedure.

Results of the logistic regression are displayed in Table 4. The variables retained in the final multivariate model for FoRP profile in cardiac patients included age, comorbidity, illness uncertainty, and cardiac distress. The model fit was significant (*X*^2^ = 210.45, *p* = <0.001), and the model accounted for a large portion of variance in FoRP levels (McFadden’s pseudo-R^2^ = 0.44).

## 4. Discussion

The present study builds upon our previous findings that FoRP is highly prevalent among people living with heart disease, spanning across a range of concerns and responses (Clarke et al., 2024b). Across all seven domains of cardiac-FoRP investigated, endorsement was on average greater than 50%. Not only were cardiac-FoRP-related issues prevalent, but often severe, being rated as ‘extremely’ concerning for up to one in five cardiac patients. These findings highlight cardiac-FoRP as a common and concerning issue.

In terms of the nature of fears experienced, the most common were unsurprisingly ‘health concerns’, which are central to the construct of FoRP, specifically fears of declining health and functioning, having another cardiac event, or the heart condition worsening. Concerns relating to oneself, such as becoming unable to engage in activities, losing control, and becoming unable to cope, were also extremely common. Concerns in both the ‘health’ and ‘self’ domains were endorsed by over three-quarters of our sample. Interpersonal concerns, such as those about others being unable to cope, treatment concerns, such as needing more procedures or medications, and role concerns, such as being unable to fulfill roles at home or work, were less common; however, they were still endorsed by over half of the sample. However, the issues that caused the most severe concern were not necessarily the most common. These were found in the domains of ‘self’ and ‘interpersonal’ concerns, where, on average, one in five participants experienced an extreme amount of concern.

Hyperawareness was both the most common and the most severe response to FoRP. These behaviors were endorsed by over 80% of the sample, with 25% of those reporting being hyperaware a lot of the time. Avoidance behaviors appeared to be less relevant, although they were still endorsed on average in over half the sample. The relatively lower rate of avoidance behaviors may reflect the wide range of endorsement within this domain, spanning from 82% avoiding stressful situations to only 11% avoiding medical appointments and check-ups. In terms of hyperawareness, many participants reported feeling overly aware of sensations in their body and worried that when they had physical sensations such as chest discomfort, increased heart rate, or shortness of breath, their cardiac disease was progressing or that they were having another event. Our previous research identified a cyclical pattern in which these concerns play a critical role in both the activation and perpetuation of cardiac-FoRP. Physical sensations such as chest tightness, rapid heart rate, and breathing difficulties are common consequences of both cardiac illnesses and fear responses and, as such, FoRP can act as both a source and a consequence of such sensations. Paired with increased vigilance to such sensations, many patients describe a distressing pattern whereby physical sensations and FoRP cyclically exaggerate each other (Clarke et al., 2024b).

Overall, the endorsement rates identified in the current study were higher than those identified in our previous research, where the average endorsement across a range of cardiac-FoRP concerns was 41% (Clarke et al., 2024b). This difference may be due to the earlier rate being calculated using responses to only 12 items that were designed for use in a broader investigation of cardiac distress. The current study used 44 items designed specifically for the purpose of investigating cardiac-FoRP and were tested in a larger and more diverse sample. By utilizing a comprehensive and specific research methodology, we identified a range of highly prevalent emotional issues for cardiac patients that may have been overlooked using a more general measure of FoRP that was not targeted for cardiac patient concerns nor validated in this population.

People living with heart disease were clustered into either high (26%), moderate (41%), or low (33%) cardiac-FoRP. Similar results have been identified in cancer research, where a cluster analysis identified a low- and high-fear group (Calderon et al., 2024). Our results differed in the identification of a moderate-FoRP group, which encompassed the majority of participants. The earlier analysis was informed using the six items of the Cancer Worry Scale (Custers et al., 2018), while the current study used 44 items across seven pre-determined domains of FoRP. While no clusters emerged based on different levels of endorsement between the seven domains, the use of this more nuanced and multidimensional representation of FoRP may underlie the disparity between the number of clusters identified in the present study of cardiac patients and previous studies of cancer patients.

The current study identified patient characteristics associated with elevated cardiac-FoRP. These include being younger, female, not having health insurance, having a mid-level (trade) education, having no close confidante, experiencing moderate-to-severe financial strain, having work impacts, having a comorbid health condition(s), and having a history of a mental health condition. Many of these factors, including younger age, mental health history, lack of a close confidante, comorbidity, financial strain, and low socio-economic indicators, such as being uninsured, have previously been identified as risk factors for anxiety, depression, and cardiac distress (Jackson et al., 2022b; Murphy et al., 2020, 2014), confirming these as important ‘red flags’ for poor post-event psychosocial functioning. Participants with heart rhythm disturbance disorders, heart failure, and ‘other’ cardiac disorders also demonstrated elevated FoRP. This suggests that disorders with more chronic profiles, as well as less common conditions such as SCAD and Takosubo cardiomyopathy, may harbor higher risks of FoRP.

Our results are similar to findings in cancer research where systematic review and metanalytic data demonstrate that younger and female patients with increased psychological morbidity are at most risk of clinically significant fear of cancer recurrence (Lebel et al., 2020; Luigjes-Huizer et al., 2022; Pang & Humphris, 2021; Smith et al., 2020). However, we did not find gender differences in the multivariate analysis, which may be due to a lower incidence of sex-specific cardiac conditions compared to cancers. It is not surprising that younger age and comorbidity were highlighted as unique predictors of cardiac-FoRP in the multivariate model: younger patients are at greater risk of significant life disruptions from their disease progressing or recurring, while those with comorbidities have greater threat of disease progression due to their multiple and complex health conditions.

As cardiac-FoRP increased, so did symptoms of anxiety, depression, PTSD, cardiac distress, and illness uncertainty. This demonstrates the strong association between cardiac-related fear and these well-established psychological constructs measured using validated instruments. Cardiac distress and illness uncertainty were retained in the multivariate model, highlighting them as unique correlates of cardiac-FoRP. Fears relating to the future are a core component of the cardiac distress framework (Jackson et al., 2022a, 2022b), and FoRP has been suggested as a critical component of how illness uncertainty is experienced in cardiac patients (Clarke et al., 2022), so these associations are not surprising. While psychological morbidity in cancer research has generally been reflective of conventional psychiatric outcomes (e.g., anxiety, depression, and PTSD), the current study demonstrated that disease-focused outcomes of distress and uncertainty outperformed other psychiatric outcomes in predicting higher levels of FoRP. In terms of the metacognitions investigated, negative beliefs about worry emerged as a stronger correlate of increasing cardiac-FoRP than did either positive beliefs about worry or need to control thoughts. This finding highlights negative beliefs about worry (e.g., my worrying is dangerous for me) as an important target for future metacognitive interventions.

The present findings have direct clinical implications for the identification of patients at risk of high cardiac-FoRP. Health professionals should be aware of the key risk factors for higher cardiac-FoRP, paying particular attention to patients who are younger, have a comorbid health condition(s), and those who express uncertainty or distress surrounding their heart health. Due to the association between FoRP and distress, the Cardiac Distress Inventory-Short Form (CDI-SF) (Le Grande et al., 2023) can be used to screen for emotional distress in cardiac patients and to open a discussion regarding FoRP and emotional support needs more broadly. Importantly, health professionals cannot assume the likelihood of high FoRP based on conventional prognostic risk factors, given that medical characteristics such as time since event, re-events or recurrent procedures, and cardiac risk factors were not associated with elevated FoRP.

The current findings also have implications regarding support options for cardiac patients, as the results suggest that the current systems in place to support cardiac patients are not adequately addressing FoRP. There was no association between cardiac-FoRP and cardiac rehabilitation attendance or receiving mental health support following a cardiac event or diagnosis. There was also no indication that cardiac-FoRP improves on its own with time. While our previous research found no differences in cardiac-FoRP endorsement rates after the first year (Clarke et al., 2024b), the current data suggest this may be true over a much longer period of time. In oncology, specific psychosocial interventions have been established as efficacious in improving FoRP (Kang & Yu, 2023; Tauber et al., 2019). Such interventions could be adapted for use in cardiac patients.

The current study also has implications regarding the measurement of cardiac-FoRP. The findings highlight a range of highly prevalent issues relating to cardiac-FoRP that may be overlooked if using a more general measure of FoRP. This underscores the importance of using relevant items developed specifically for cardiac patients. This is particularly pertinent when considering that almost all research into cardiac-FoRP to date has used general measures that have not been validated for use in cardiac patients (Clarke et al., 2024b). More recently, a transdiagnostic measure of FoRP has been developed and validated in a range of chronic illnesses, including cardiac patients (Sharpe et al., 2023). Moreover, a cardiac-disease-specific FoRP measure is currently in development (Clarke et al., 2024a), using the same item pool presented in the current study. This new measure will be important not only for facilitating future research, but also for use in practice to identify patients in most need of support, particularly since patients may feel reluctant to raise such concerns on their own in fear of appearing ungrateful or damaging their relationship with their clinician (Ozakinci et al., 2018).

The current study has some limitations that should be acknowledged. First, while the study identified key risk factors for cardiac-FoRP, it did not assess negative outcomes associated with these fears. Longitudinal designs should be enlisted to explore this, both in relation to cardiac risk and psychosocial wellness. Second, while the results identified cardiac distress and illness uncertainty as unique predictors of FoRP, further investigation is required to fully understand the relationship between these factors, particularly whether one of these constructs mediates the relationship between the others. This would inform the development of interventions targeted towards the underlying relationship. Third, while the current study included a wide range of cardiac presentations, it did not establish whether risk factors differed between diagnoses. This was not possible due to the large number of cardiac events, conditions, and procedures, often coexisting, rendering it infeasible to split the sample. Future research could investigate differences between cardiac conditions, particularly in relation to those with rare conditions and chronic presentations, who presented with higher levels of FoRP than those with more common conditions with acute presentations. Lastly, due to the convenience sampling, we relied on participants’ self-reports without independent verification, including information regarding participants’ cardiac conditions. Further, future research may wish to assess the generalizability of these results in a consecutive case series.

## 5. Conclusions

The findings of the current study contribute to our understanding of the nature of and risk factors for cardiac-FoRP. The results established that concerns and behaviors related to cardiac-FoRP are highly endorsed, specifically in the domains of health concerns and hyperawareness. Three fear clusters were identified—low, moderate, and high. The most important risk factors for higher cardiac-FoRP were being younger, having a comorbid health condition(s), and having higher levels of cardiac distress and illness uncertainty. The next steps for research are to establish more specific and validated methods with which to quantify cardiac-FoRP and identify patients in need of support. Tailored cardiac-FoRP interventions should be adapted from those available for cancer patients so practitioners can offer appropriate support to those in need.

## Figures and Tables

**Table 2:** Differences in participant characteristics across FoRP profiles (ANOVA).

	Low-FoRP Group				Moderate-FoRP Group				High-FoRP Group					
Variable	*n*	*M*	*SD*		*n*	*M*	*SD*		*n*	*M*	*SD*	*X* ^2^	*p*	*ε* ^2^
Age	79	67.95	10.52		100	64.63	10.82		62	59.97	11.31	17.41	**<0.001**	0.07
Time since														
First event/diagnosis	77	66.15	111.92		93	72.92	114.31		59	68.87	93.93	2.24	0.33	0.01
Recent event/diagnosis	75	26.94	45.70		94	19.78	34.23		56	18.77	26.49	0.64	0.73	0.00
Depression	73	3.15	3.39		95	7.11	5.02		58	13.71	5.91	100.65	**<0.001**	0.45
Anxiety	73	2.10	2.90		95	5.53	4.97		58	9.90	5.38	75.40	**<0.001**	0.34
PTSD	73	7.03	7.99		97	16.43	11.15		58	33.97	17.86	91.92	**<0.001**	0.40
Illness uncertainty	73	41.05	11.10		96	54.73	11.86		58	66.76	14.93	85.77	**<0.001**	0.38
Cardiac distress	75	3.00	3.39		98	9.32	5.84		59	19.61	7.49	132.39	**<0.001**	0.57
Metacognitions														
Positive beliefs	73	7.85	3.00		95	9.19	3.66		57	10.11	4.47	13.31	**0.001**	0.06
Negative beliefs	73	8.64	2.93		95	11.46	4.30		57	13.86	4.82	44.49	**<0.001**	0.20
Need for control	73	8.68	2.70		95	9.61	2.92		57	11.09	3.96	17.05	**<0.001**	0.08

Bold indicates where *p* < 0.05.

**Table 3:** Post hoc comparison of participant characteristics across FoRP profiles (ANOVA).

	Low vs. High FoRP		Low vs. Moderate FoRP		Moderate vs. High FoRP	
	W	*p*	W	*p*	W	*p*
Age	5.77	**<0.001**	2.98	0.09	3.59	**0.03**
Anxiety	−13.37	**<0.001**	−7.78	**<0.001**	−7.11	**<0.001**
Depression	−12.55	**<0.001**	−8.96	**<0.001**	−9.29	**<0.001**
PTSD	−11.60	**<0.001**	−8.96	**<0.001**	−8.70	**<0.001**
Cardiac distress	−13.37	**<0.001**	−11.16	**<0.001**	−10.83	**<0.001**
Illness uncertainty	−11.43	**<0.001**	−9.51	**<0.001**	−6.79	**<0.001**
Positive beliefs	−4.75	**0.002**	−4.08	**0.01**	−1.48	0.55
Negative beliefs	−8.81	**0.001**	−6.53	**<0.001**	−4.32	**0.006**
Need for control	−5.64	**<0.001**	−3.37	**0.046**	−3.28	0.05

W = Wilcoxon rank sum test statistics. Bold indicates where *p* < 0.05.

**Table 4:** Ordinal logistic regression for factors associated with cardiac-FoRP.

		95% CI		*p*
Variable	Odds Ratio	Low	High	
Age	1.03	1.00	1.07	**0.04**
Female gender	0.65	0.34	1.28	0.26
Education				
Secondary—University	1.65	0.63	4.35	0.30
Trade—University	0.64	0.29	1.43	0.28
No health insurance	1.00	0.44	2.27	0.95
No close confidant	1.28	0.50	3.29	0.61
Moderate-severe financial strain	0.80	0.38	1.66	0.56
History of mental ill health	0.79	0.39	1.58	0.51
Work impacted by cardiac health	1.29	0.66	2.52	0.45
>one cardiac event/diagnosis	1.11	0.56	2.21	0.76
Comorbidity	0.32	0.15	0.66	**0.003**
Anxiety symptoms	1.02	0.91	1.15	0.70
Depression symptoms	1.07	0.96	1.20	0.21
PTSD symptoms	0.97	0.94	1.01	0.16
Cardiac distress symptoms	0.77	0.70	0.84	**<0.001**
Illness uncertainty	0.95	0.93	0.98	**0.001**
Positive beliefs	0.97	0.88	1.08	0.59
Negative beliefs	1.00	0.89	1.13	0.99
Need for control	0.93	0.81	1.05	0.25

Bold indicates where *p* < 0.05.

## Data Availability

The data presented in this study are available on reasonable request from the corresponding author.

## Supplementary Materials

The following supporting information can be downloaded at https://www.mdpi.com/article/10.3390/bs15040479/s1, Table S1. Cardiac conditions, events, and procedures specified as other. Table S2. Response rates for FoRP items.
