# Supplementary material for: Fear of Recurrence and Progression in People with Heart Disease: Risk Factors and Implications for Emotional Support
Source: Behav Sci (Basel). 2025 Apr 6;15(4):479. doi: 10.3390/bs15040479 (PMC12024251; doi:10.3390/bs15040479)
Supplement: Supplementary file 1 [file behavsci-15-00479-s001.zip › behavsci-3512224-supplementary.pdf]

Table S1. Cardiac conditions, events, and procedures specified as other.

**Condition/event/procedure**	**n**
Pacemaker*	3
Cardiomyopathy	3
Coronary artery spasm	2
Left ventricular outflow tract obstruction	1
Aortic aneurism	2
Ascending aortic aneurysm repair	1
Aortic dissection	1
Repaired Coarctation of Aorta	1
Atrial myxoma	1
Cardiac amyloidosis	1
Postural orthostatic tachycardia syndrome (POTS)	1
Pericarditis	1
Pericardiectomy	1
Left/right bundle branch block	2
Atrioventricular block*	1
Heart murmur	1
David Procedure	1
Tricuspid and Mitral valve regurgitation	1

*** = same participant.** Total n for “other” category was n =24.

Table S2. Responses rates for FoRP items.

**Fears**				
	Extremely (%)	*Moderately (%)*	*Slightly (%)*	*Not at all (%)*
Health				
Your general health and functioning declining.	22	41	24	13
Having another heart event.	17	33	36	14
Your condition getting worse.	13	32	37	19
Developing other medical problems.	15	34	33	19
Dying.	13	27	32	27
Physical activity leading to another heart event.	9	25	37	29
Interpersonal				
Other people being unable to cope if something happens to you.	20	23	32	26
Becoming a burden to your family.	22	27	25	27
Becoming more withdrawn from friends.	16	18	33	33
Becoming socially isolated.	15	14	32	40
Becoming lonely.	13	18	27	42
Your condition impacting your intimate relationships.	12	23	19	45

Losing capacity for sexual activity.	9	21	21	50
Self				
Becoming unable to engage in activities you enjoy.	25	32	28	15
Losing control over your life.	20	25	35	20
Becoming unable to cope effectively.	13	25	40	22
Never getting back to the person you used to be.	27	26	24	22
Being unable to plan for the future.	18	21	36	25
Ageing.	9	31	33	27
Becoming less of a person.	15	15	29	41
Treatment				
Needing more procedures or surgery.	16	29	32	23
Needing to take more medications.	19	24	33	24
Needing to go back to hospital.	13	24	33	29
Being unable to get help on time.	13	24	31	31
Not having access to the health care you might need.	10	17	32	40
Being unable to afford future medical treatments.	12	16	29	43
Receiving permanent scarring from future heart surgeries or removal of veins.	5	11	20	65
Roles				
Becoming unable to fulfil your roles at home.	13	26	34	26
Becoming unable to support yourself financially.	17	18	27	38
Becoming unable to work.	15	14	21	50
Becoming unable to fulfil your roles at work.	12	16	12	60
**Behaviours**				
	A lot of the time (%)	*Some of the time (%)*	*A little of the time (%)*	*None of the time (%)*
Avoidance				
Avoid stressful situations.	19	26	37	18
Avoid thinking about your heart condition.	11	28	31	30
Avoid activities that make your heart beat faster.	13	27	29	31
Avoid physical exertion.	15	24	29	31
Avoid going far from home.	12	18	20	50
Avoid travelling far from your cardiac care team.	6	16	17	61
Avoid being alone.	5	10	18	67
Avoid medical appointments or check-ups.	1	3	7	89

Hyperawareness				
Feel worried that your condition is getting worse when you notice changes in your body, such as feeling more fatigued, short of breath, or retaining more fluid	21	29	33	17
Feel overly aware of sensations in your body.	21	25	37	17
Feel worried that you are having another event when you have chest discomfort, or when your heartbeat is fast or irregular	16	32	35	17
Monitor your heart rate.	22	29	30	19
Feel overly aware of your heart in your chest.	20	24	32	23
